# Early subclinical left ventricular dysfunction after surgical menopause: a prospective speckle-tracking echocardiography study

**DOI:** 10.1186/s12905-026-04672-z

**Published:** 2026-07-09

**Authors:** Doğukan Yazıcı, Yunus Emre Özbebek, Ülkü Gürbüz Özbebek, Batıkan Yazıcı, Tuğba Kolomuç Gayretli, Hüseyin Levent Keskin, Hatice Tolunay

**Affiliations:** 1 Department of Cardiology, Dr. Burhan Nalbantoğlu State Hospital, Nicosia, Northern Cyprus Cyprus; 2 Department of Cardiology, Ankara Etlik City Hospital, Ankara, Türkiye; 3 Department of Obstetrics and Gynecology, Sincan Training and Research Hospital, Ankara, Türkiye; 4 Department of Cardiology, Ankara Bilkent City Hospital, Ankara, Türkiye; 5 Department of Obstetrics and Gynecology, Ankara Etlik City Hospital, Ankara, Türkiye; 6 Department of Obstetrics and Gynecology, Ufuk University Faculty of Medicine, Ankara, Türkiye

**Keywords:** Surgical menopause, Natural menopause, Left ventricular global longitudinal strain, Speckle-tracking echocardiography, Subclinical left ventricular dysfunction, Estrogen deficiency

## Abstract

**Background:**

Menopause is associated with adverse cardiovascular changes, while surgical menopause results in a more abrupt loss of ovarian function than natural menopause. However, its early impact on subclinical left ventricular systolic function remains insufficiently defined. This study aimed to compare longitudinal changes in left ventricular global longitudinal strain (LV GLS) between women with recently confirmed natural menopause and women undergoing surgical menopause.

**Methods:**

This prospective observational study included 42 women with recently confirmed natural menopause and 39 perimenopausal women scheduled to undergo bilateral oophorectomy for benign gynecological indications, who subsequently constituted the surgical menopause group. In the surgical menopause group, baseline echocardiography was performed preoperatively during the perimenopausal period, whereas follow-up examinations were performed at 1 and 6 months after bilateral oophorectomy. Transthoracic echocardiography and two-dimensional speckle-tracking analysis were performed at baseline and at 1- and 6-month follow-up visits. Longitudinal changes in LV GLS were evaluated using a linear mixed-effects model adjusted for age, including time × menopause group and time × age interaction terms.

**Results:**

Women in the surgical menopause group were older than those in the natural menopause group (49.59 ± 2.36 vs. 46.07 ± 2.23 years, *p* < 0.001), whereas baseline left ventricular ejection fraction and LV GLS were comparable. At 1 month, LV GLS was significantly less negative in the surgical menopause group than in the natural menopause group (− 16.88 ± 1.25% vs. −17.60 ± 1.64%, *p* = 0.030). At 6 months, LV GLS remained numerically less negative in the surgical menopause group, but the between-group difference was not statistically significant (− 16.09 ± 1.10% vs. −16.64 ± 1.55%, *p* = 0.070). In the age-adjusted linear mixed-effects model, the time × surgical menopause interaction remained significant at 1 month (β = 0.50, 95% confidence interval [CI]: 0.07–0.92, *p* = 0.024) but not at 6 months (β = 0.18, 95% CI: −0.25 to 0.61, *p* = 0.400).

**Conclusion:**

Surgical menopause was associated with a more pronounced early deterioration in LV GLS at 1 month after adjustment for age, despite preserved left ventricular ejection fraction. However, an independent between-group difference was not demonstrated at 6 months. LV GLS may serve as an adjunctive tool for the early cardiovascular monitoring of women undergoing surgical menopause, although its long-term prognostic value requires validation in larger studies with longer follow-up.

## Introduction

Cardiovascular disease remains a leading cause of morbidity and mortality among women, and the menopausal transition represents a critical period during which cardiovascular risk begins to increase [1]. The decline in endogenous estrogen levels during menopause has been associated with unfavorable changes in vascular function, lipid metabolism, insulin resistance, body composition, and blood pressure regulation. Therefore, menopause is increasingly recognized not only as a reproductive milestone but also as an important cardiometabolic transition period [2, 3].

Surgical menopause, usually resulting from bilateral oophorectomy with or without hysterectomy, differs from natural menopause because of the abrupt cessation of ovarian hormone production [4]. Compared with the gradual hormonal changes observed during natural menopause, surgical menopause may cause a more sudden estrogen-deficient state [5]. Previous studies have suggested that women undergoing early bilateral oophorectomy may have increased cardiovascular risk and mortality, particularly when estrogen deficiency occurs before the expected age of natural menopause [6, 7]. However, the early myocardial consequences of surgical menopause remain less clearly defined.

Conventional echocardiographic parameters, particularly left ventricular ejection fraction, are widely used to evaluate systolic function. Nevertheless, LVEF may remain within the normal range despite early myocardial impairment. Left ventricular global longitudinal strain, derived from two-dimensional speckle-tracking echocardiography, is a sensitive marker of subclinical systolic dysfunction and can detect subtle changes in myocardial deformation before a reduction in LVEF becomes apparent [8, 9]. Although the cardiovascular effects of menopause have been widely studied, limited data are available regarding serial changes in LV GLS after surgical menopause compared with natural menopause. Identifying early subclinical myocardial dysfunction in this population may help clarify the cardiovascular impact of abrupt estrogen deprivation and may support closer cardiovascular surveillance in women undergoing surgical menopause.

Therefore, this prospective echocardiographic follow-up study was specifically designed to compare longitudinal changes in LV GLS between women with recent natural menopause and those with recent surgical menopause during the early postmenopausal period. We hypothesized that LV GLS deterioration would be more pronounced in the surgical menopause group despite preserved LVEF. A premenopausal control group was not included because the primary objective was to compare the early myocardial effects of the two different modes of menopausal transition.

## Methods

### Study design and population

This prospective observational study was conducted between February 2025 and August, 2025 at our Hospital. Women with recently confirmed natural menopause and perimenopausal women scheduled to undergo bilateral oophorectomy for benign gynecological indications were screened for eligibility. Women undergoing bilateral oophorectomy subsequently constituted the surgical menopause group. In the surgical menopause group, the baseline echocardiographic examination was performed during the preoperative evaluation before bilateral oophorectomy. Follow-up echocardiographic examinations were performed approximately 1 and 6 months after surgery. In the natural menopause group, echocardiographic examinations were performed at enrollment and at the corresponding 1- and 6-month follow-up visits. The 1-month assessment was selected to evaluate early changes following the menopausal transition, whereas the 6-month assessment was intended to determine whether these changes persisted or evolved during short-term follow-up. No formal a priori sample size calculation was performed because the study was designed as a prospective exploratory investigation, and eligible participants were consecutively enrolled during the predefined study period.

The study was conducted in accordance with the principles of the Declaration of Helsinki. The study protocol was approved by the Ethics Committee of Ankara Etlik City Hospital (Approval No: AEŞH-BADEK-2025-0174; approval date: 29 January 2025). All participants were informed about the study protocol, and written informed consent was obtained before inclusion.

### Inclusion and exclusion criteria

Women were eligible for inclusion if they either had natural menopause confirmed within the preceding month after fulfillment of the 12-month amenorrhea criterion or were perimenopausal and scheduled to undergo bilateral oophorectomy for a benign gynecological indication, with preserved left ventricular ejection fraction on baseline transthoracic echocardiography.

Patients were excluded if they had a history of atherosclerotic cardiovascular disease; rhythm or conduction disorders, including atrial fibrillation; chronic use of medications that could influence myocardial contractility or heart rate, including beta-blockers, non-dihydropyridine calcium-channel blockers, and class I or III antiarrhythmic agents; current or planned hormone replacement therapy; systemic conditions requiring medical treatment, including hypertension, diabetes mellitus, hyperlipidemia, anemia, renal failure, or thyroid disease; moderate or severe valvular heart disease; active or previous pericarditis; malignancy; active infection; inability to provide informed consent; left ventricular ejection fraction below 50%; or regional wall motion abnormalities on transthoracic echocardiography.

Patients with inadequate echocardiographic image quality for speckle-tracking analysis and patients who developed acute coronary syndrome during follow-up were also excluded from the final analysis.

### Definitions of menopause groups

Natural menopause was defined as at least 12 consecutive months of spontaneous amenorrhea without another physiological or pathological cause and without a history of bilateral oophorectomy. Because natural menopause can only be confirmed retrospectively, the timing of menopause onset was estimated according to the date of the final menstrual period. Women in the natural menopause group were enrolled within 1 month after fulfillment and clinical confirmation of the 12-month amenorrhea criterion.

Surgical menopause was defined as the permanent cessation of ovarian function following bilateral oophorectomy. All women in the surgical menopause group underwent bilateral oophorectomy, with or without concomitant hysterectomy and/or salpingectomy, for benign gynecological indications. Women who underwent unilateral oophorectomy or hysterectomy without bilateral oophorectomy were not included in the surgical menopause group.

### Echocardiographic assessment

Transthoracic echocardiographic examinations were performed at baseline and at the 1- and 6-month follow-up visits using the same commercially available ultrasound system (Vivid E95, GE Healthcare, Horten, Norway) equipped with a phased-array transducer. Echocardiographic measurements were obtained in accordance with the recommendations of the European Association of Cardiovascular Imaging [10].

Standard echocardiographic parameters included left ventricular ejection fraction, left ventricular end-diastolic diameter, left ventricular end-systolic diameter, lateral and septal e′ velocities, E/e′ ratio, tricuspid annular plane systolic excursion, left atrial volume index, and aortic flow velocity. Left ventricular ejection fraction was assessed using conventional two-dimensional echocardiographic methods. Diastolic function parameters were measured using pulsed-wave and tissue Doppler imaging where appropriate.

### Speckle-tracking echocardiography

Left ventricular global longitudinal strain was assessed using two-dimensional speckle-tracking echocardiography from the apical two-chamber, three-chamber, and four-chamber views. Two-dimensional grayscale images were acquired at a frame rate of 50–80 frames per second and digitally stored for analysis. Strain analysis was performed directly on the Vivid E95 ultrasound system using its integrated vendor-specific software. Endocardial borders were manually traced, and myocardial tracking quality was visually inspected. The region of interest and segments with inadequate tracking were manually adjusted when necessary. LV GLS was calculated as the average peak systolic longitudinal strain derived from the three standard apical views, and bull’s-eye maps were generated.

All speckle-tracking analyses were performed by a single experienced cardiologist who was blinded to the participants’ menopause group and clinical data. Echocardiographic images were anonymized, and the observer did not have access to measurements obtained at the other study visits during the analysis.

LV GLS values were reported as negative values. More negative LV GLS values indicate better longitudinal systolic function, whereas less negative values indicate worsening longitudinal systolic function.

### Statistical analysis

The normality of continuous variables was assessed using the Shapiro-Wilk test. Normally distributed continuous variables were presented as mean ± standard deviation, whereas non-normally distributed variables were presented as median values or according to data availability. Categorical variables were expressed as numbers and percentages. Between-group comparisons were performed using the independent samples t-test for normally distributed continuous variables and the Mann-Whitney U test for non-normally distributed variables. Categorical variables were compared using Fisher’s exact test.

Longitudinal changes in LV GLS and the interaction between time and menopause type were evaluated using a linear mixed-effects model. The model included time, menopause group, age, and the time × group and time × age interaction terms as fixed effects. Age was centered at the overall sample mean before inclusion in the model. Patient identity was included as a random intercept to account for repeated measurements within individuals. The model was estimated using restricted maximum likelihood. Post-hoc pairwise comparisons were adjusted using the Tukey method, and degrees of freedom were calculated using the Kenward–Roger method. Model estimates are presented as β coefficients with 95% confidence intervals. A two-sided p value of < 0.05 was considered statistically significant. All statistical analyses were performed using R statistical software, version 4.3.

## Results

### Baseline characteristics

A total of 81 consenting women with adequate echocardiographic image quality were enrolled and included in the final analysis, comprising 42 women in the natural menopause group and 39 women in the surgical menopause group. All 81 participants completed the baseline, 1-month, and 6-month assessments, and no participant was lost to follow-up. The exact numbers of women who declined participation or were excluded before enrollment because of inadequate echocardiographic image quality were not systematically recorded. Baseline demographic and laboratory characteristics are summarized in Table 1. The mean age was significantly higher in the surgical menopause group than in the natural menopause group (49.59 ± 2.36 years vs. 46.07 ± 2.23 years, *p* < 0.001). Body surface area and SCORE2 values were comparable between the groups. Hemoglobin and white blood cell count also did not differ significantly between the groups.

**Table 1:** Baseline characteristics of the study population according to menopause type

Variable	Natural menopause (*n* = 42)	Surgical menopause (*n* = 39)	*p* value
Age, years	46.07 ± 2.23	49.59 ± 2.36	< 0.001
Body surface area, m²	1.90 ± 0.17	1.96 ± 0.11	0.063
SCORE2	4.04 ± 0.70	4.04 ± 0.69	0.984
Hemoglobin, g/dL	12.8 ± 1.31	12.5 ± 2.00	0.251
White blood cell count, ×10³/µL	7.5 ± 1.0	7.8 ± 2.0	0.356

Data are presented as mean ± standard deviation

*SCORE2* Systematic Coronary Risk Evaluation 2

### Serial echocardiographic findings

Serial echocardiographic parameters according to menopause type are presented in Table 2. At baseline, conventional left ventricular systolic function was preserved in both groups, and there was no significant difference in LVEF between the natural menopause and surgical menopause groups (60.43 ± 1.64% vs. 60.33 ± 1.30%, *p* = 0.774). Baseline LV GLS values were also comparable between groups (-18.25 ± 1.69% vs. -18.22 ± 1.45%, *p* = 0.917).

**Table 2:** Serial echocardiographic findings according to menopause type

Parameter	Time point	Natural menopause (*n*=42)	Surgical menopause (*n*=39)	*p* value
LVEF, %	Baseline	60.43 ± 1.64	60.33 ± 1.30	0.774
	1 month	60.50 ± 1.61	60.33 ± 1.30	0.612
	6 months	60.43 ± 1.64	60.36 ± 1.32	0.837
LVEDD, cm	Baseline	4.70 ± 0.15	4.70 ± 0.19	0.740
	1 month	4.70 ± 0.15	4.71 ± 0.18	0.891
	6 months	4.70 ± 0.15	4.69 ± 0.18	0.937
LVESD, cm	Baseline	2.90 ± 0.22	2.93 ± 0.31	0.172
	1 month	2.91 ± 0.26	2.86 ± 0.42	0.458
	6 months	2.90 ± 0.29	2.88 ± 0.42	0.858
Lateral e′, cm/s	Baseline	14.07 ± 2.42	13.10 ± 1.72	0.055
	1 month	13.38 ± 2.05	14.33 ± 3.21	0.151
	6 months	13.25 ± 2.24	12.85 ± 1.59	0.343
Septal e′, cm/s	Baseline	8.41 ± 1.59	8.38 ± 1.57	0.896
	1 month	8.40 ± 1.79	7.46 ± 1.57	0.014
	6 months	7.14 ± 1.60	7.16 ± 1.46	0.279
E/e′	Baseline	5.41 ± 1.26	5.02 ± 0.74	0.113
	1 month	5.49 ± 1.21	5.08 ± 0.69	0.069
	6 months	5.49 ± 1.19	5.05 ± 0.71	0.072
TAPSE, mm	Baseline	26.40 ± 2.03	25.80 ± 2.52	0.305
	1 month	26.33 ± 1.97	25.79 ± 2.43	0.244
	6 months	25.83 ± 1.73	25.85 ± 2.25	0.275
LAVI, mL/m²	Baseline	19.17 ± 2.05	19.97 ± 1.70	0.059
	1 month	19.17 ± 2.06	20.19 ± 1.71	0.043
	6 months	20.48 ± 2.03	20.87 ± 1.75	0.061
LV GLS, %	Baseline	-18.25 ± 1.69	-18.22 ± 1.45	0.917
	1 month	-17.60 ± 1.64	-16.88 ± 1.25	0.030
	6 months	-16.64 ± 1.55	-16.09 ± 1.10	0.070
Aortic velocity, m/s	Baseline	1.27 ± 0.13	1.27 ± 0.13	0.735
	1 month	1.25 ± 0.11	1.26 ± 0.11	0.674
	6 months	1.24 ± 0.11	1.25 ± 0.11	0.767

Data are presented as mean ± standard deviation

More negative LV GLS values indicate better longitudinal systolic function. Less negative LV GLS values indicate worsening longitudinal systolic function

*LAVI* Left atrial volume index, *LVEDD* Left ventricular end-diastolic diameter, *LVEF* Left ventricular ejection fraction, *LVESD* Left ventricular end-systolic diameter, *LV GLS* Left ventricular global longitudinal strain, *TAPSE* Tricuspid annular plane systolic excursion

At the 1-month follow-up, LVEF remained similar between the two groups (60.50 ± 1.61% vs. 60.33 ± 1.30%, *p* = 0.612). However, LV GLS was significantly less negative in the surgical menopause group compared with the natural menopause group (-16.88 ± 1.25% vs. -17.60 ± 1.64%, *p* = 0.030), indicating more pronounced impairment in longitudinal systolic function. In addition, septal e′ velocity was significantly lower in the surgical menopause group (7.46 ± 1.57 cm/s vs. 8.40 ± 1.79 cm/s, *p* = 0.014), while LAVI was significantly higher in the surgical menopause group (20.19 ± 1.71 mL/m² vs. 19.17 ± 2.06 mL/m², *p* = 0.043).

At the 6-month follow-up, LVEF remained preserved and comparable between the groups (60.36 ± 1.32% vs. 60.43 ± 1.64%, *p* = 0.837). LV GLS remained numerically less negative in the surgical menopause group compared with the natural menopause group (-16.09 ± 1.10% vs. -16.64 ± 1.55%), although this difference did not reach statistical significance (*p* = 0.070). Other conventional echocardiographic parameters, including LVEDD, LVESD, lateral e′ velocity, septal e′ velocity, E/e′ ratio, TAPSE, LAVI, and aortic velocity, were not significantly different between the groups at 6 months.

### Longitudinal changes in LV GLS

**Fig. 1:**
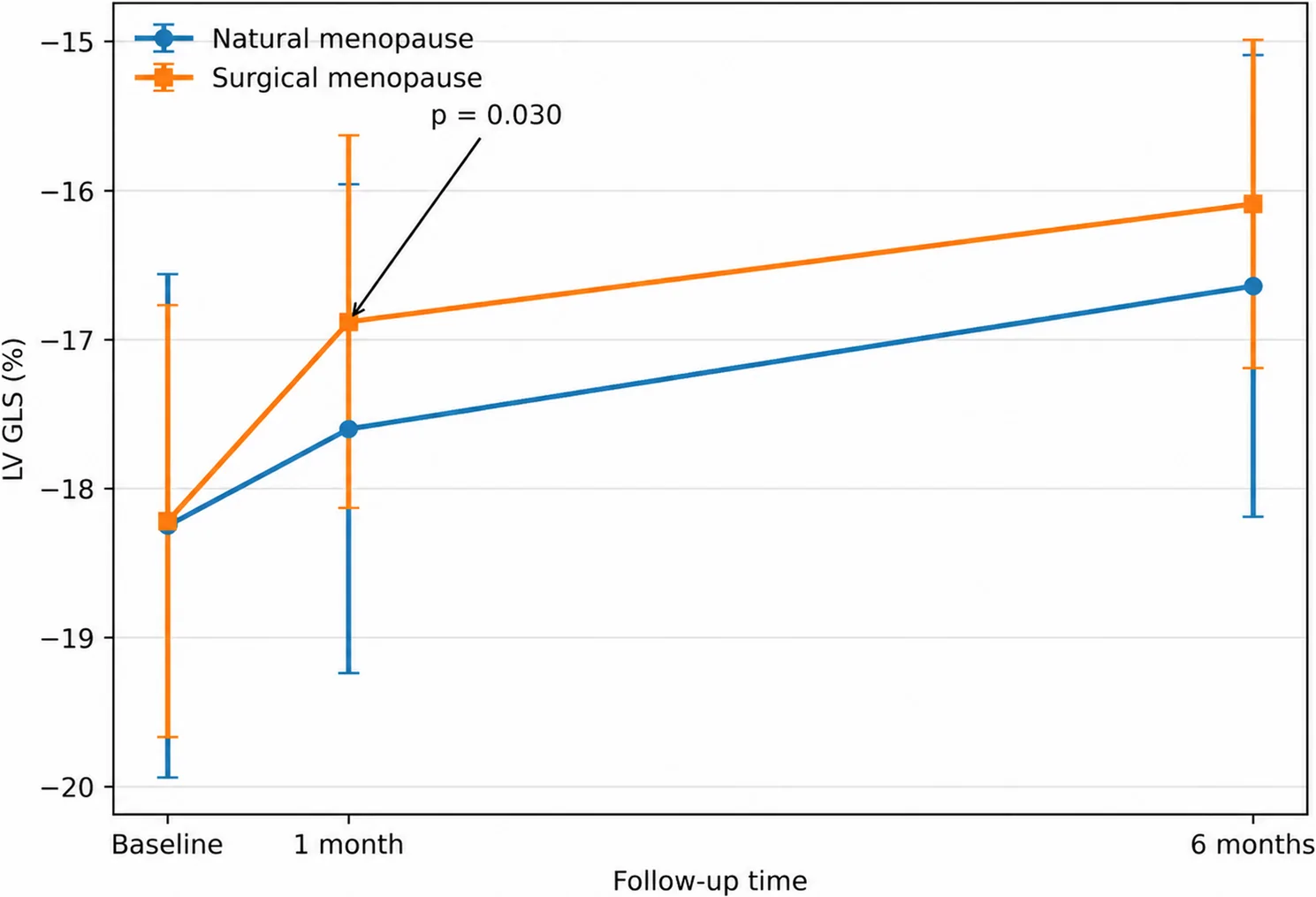
Changes in left ventricular global longitudinal strain over time according to menopause type. Values are presented as mean ± SD. LV GLS became progressively less negative during follow-up in both groups, indicating worsening longitudinal systolic function. The deterioration was more pronounced in the surgical menopause group, with a significant between-group difference at 1 month (*p* = 0.030). Less negative LV GLS values indicate worsening longitudinal systolic function. LV GLS: left ventricular global longitudinal strain

The temporal changes in LV GLS according to menopause type are shown in Fig. 1. LV GLS became progressively less negative over time in both groups, indicating worsening longitudinal systolic function. At the 1-month assessment, this deterioration was more pronounced in the surgical menopause group; however, the between-group difference was not statistically significant at 6 months.

An age-adjusted linear mixed-effects model was used to evaluate longitudinal changes in LV GLS. The model included time, menopause group, age, time × group interaction terms, and time × age interaction terms as fixed effects, with patient identity included as a random intercept. The results of the age-adjusted linear mixed-effects model are presented in Table 3. After adjustment for age, LV GLS deteriorated significantly at 1 month (β = 0.74, 95% CI: 0.48–1.01, *p* < 0.001) and at 6 months (β = 1.77, 95% CI: 1.51–2.04, *p* < 0.001) compared with baseline. The baseline difference between the natural and surgical menopause groups was not statistically significant (β = 0.14, 95% CI: −0.69 to 0.96, *p* = 0.743).

**Table 3:** Age-adjusted linear mixed-effects model for longitudinal changes in LV GLS

Effect	Estimate, β	95% CI	*p* value
Intercept	−18.30	−18.81 to − 17.80	< 0.001
Time: 1 month	0.74	0.48 to 1.01	< 0.001
Time: 6 months	1.77	1.51 to 2.04	< 0.001
Group: surgical menopause	0.14	−0.69 to 0.96	0.743
Time × group: 1 month × surgical menopause	0.50	0.07 to 0.92	0.024
Time × group: 6 months × surgical menopause	0.18	−0.25 to 0.61	0.400
Age	−0.03	−0.17 to 0.12	0.696
Time × age: 1 month	0.05	−0.02 to 0.13	0.153
Time × age: 6 months	0.10	0.02 to 0.17	0.012

The natural menopause group and baseline measurement were used as the reference categories. Age was centered at the overall sample mean before inclusion in the model. The model included time, menopause group, age, time × group interaction terms, and time × age interaction terms as fixed effects, with patient identity included as a random intercept. A positive β coefficient indicates a shift toward less negative LV GLS values, reflecting worsening longitudinal systolic function

*CI* Confidence interval, *LV GLS* Left ventricular global longitudinal strain

The time × surgical menopause interaction remained statistically significant at 1 month (β = 0.50, 95% CI: 0.07–0.92, *p* = 0.024), indicating a greater early deterioration in LV GLS in the surgical menopause group after adjustment for age. However, the corresponding interaction at 6 months was not statistically significant (β = 0.18, 95% CI: −0.25 to 0.61, *p* = 0.400).

The time × age interaction was not statistically significant at 1 month (β = 0.05, 95% CI: −0.02 to 0.13, *p* = 0.153) but was significant at 6 months (β = 0.10 per year, 95% CI: 0.02–0.17, *p* = 0.012), indicating that older age was associated with greater deterioration in LV GLS at the 6-month assessment.

### Post-hoc analysis

Post-hoc pairwise comparisons demonstrated significant changes in LV GLS across all time points within both groups. In the natural menopause group, LV GLS significantly changed from baseline to 1 month, from baseline to 6 months, and from 1 month to 6 months. Similar significant changes were observed in the surgical menopause group. Within-group pairwise comparisons demonstrated significant changes across study visits in both groups. Between-group differences in longitudinal trajectories were interpreted according to the age-adjusted mixed-effects model.

## Discussion

In this prospective echocardiographic follow-up study, we evaluated serial changes in left ventricular systolic function in women with recently confirmed natural menopause and women undergoing surgical menopause using two-dimensional speckle-tracking echocardiography. The principal finding was that women with surgical menopause exhibited a more pronounced early deterioration in LV GLS at the 1-month assessment despite preserved LVEF. This difference remained statistically significant after adjustment for age. However, the age-adjusted time × group interaction was not significant at 6 months, indicating that an independent and persistent effect of menopause type could not be demonstrated throughout the entire follow-up period.

The menopausal transition is associated with adverse cardiometabolic changes, including alterations in body composition, lipid metabolism, insulin sensitivity, endothelial function, vascular stiffness, and blood pressure regulation [1, 11]. Surgical menopause differs from natural menopause because ovarian function is lost abruptly rather than gradually, particularly following bilateral oophorectomy [4, 5]. Previous studies have associated bilateral oophorectomy and early surgical menopause with increased long-term cardiovascular risk and mortality [6, 7]. Nevertheless, the mechanisms underlying the early myocardial changes observed in the present study cannot be established. Although abrupt changes in ovarian hormone levels represent a biologically plausible explanation, estradiol, follicle-stimulating hormone, and luteinizing hormone levels were not measured. Estrogen has been associated with favorable effects on endothelial function, oxidative stress, inflammation, vascular tone, and myocardial metabolism; however, the present findings should not be interpreted as direct evidence of an estrogen-mediated myocardial effect [12].

The significant age difference between the study groups represents an important consideration. Women in the surgical menopause group were older than those in the natural menopause group, and age may influence myocardial deformation even in the presence of preserved LVEF and similar baseline LV GLS. Comparable baseline LV GLS values do not fully exclude age-related differences in subsequent myocardial trajectories. We therefore repeated the longitudinal analysis after incorporating age and time × age interaction terms into the linear mixed-effects model. The more pronounced deterioration associated with surgical menopause remained significant at 1 month but was no longer significant at 6 months. Furthermore, older age was associated with greater LV GLS deterioration at the 6-month assessment. These findings suggest that age may partly explain the later longitudinal changes and support a cautious interpretation of the unadjusted group comparisons.

At baseline, LVEF and LV GLS were comparable between the natural and surgical menopause groups. At 1 month, LV GLS was significantly less negative in the surgical menopause group, suggesting a greater early impairment in longitudinal systolic function. In contrast, the conventional between-group comparison at 6 months did not reach statistical significance, and the age-adjusted time × group interaction was also not significant. Therefore, the findings primarily support a short-term difference during the early postmenopausal period rather than a sustained independent divergence between the groups.

Previous deformation-imaging studies also suggest that ovarian aging and loss of ovarian function may be associated with subclinical myocardial impairment despite preserved conventional systolic function. Wang et al. demonstrated impaired layer-specific longitudinal and circumferential strain in newly diagnosed, untreated patients with premature ovarian insufficiency despite preserved LVEF compared with healthy controls [13]. These findings are broadly consistent with the progressive deterioration in LV GLS observed in both groups in our study. However, the present investigation extends previous cross-sectional observations by providing serial measurements across the surgical menopausal transition. Importantly, the absence of a significant age-adjusted time × group interaction at 6 months indicates that a persistent divergence between natural and surgical menopause remains uncertain.

The preservation of LVEF throughout follow-up is clinically relevant. Conventional LVEF may remain within the normal range despite early myocardial dysfunction, whereas LV GLS can identify subtle abnormalities in longitudinal myocardial deformation before overt systolic dysfunction becomes apparent [8, 9]. In the present study, LVEF remained stable in both groups, while LV GLS became progressively less negative during follow-up. These findings support the potential value of speckle-tracking echocardiography for identifying early subclinical myocardial changes that may not be detected using conventional systolic parameters.

At the 1-month assessment, septal e′ velocity was lower and LAVI was higher in the surgical menopause group. These findings may reflect transient differences in diastolic function or loading conditions during the early postoperative period. However, neither difference persisted at 6 months, and heart rate and blood pressure at the time of echocardiographic acquisition were not systematically recorded. Therefore, these secondary findings should be interpreted cautiously and cannot be definitively attributed to menopause-related myocardial changes.

An additional consideration is that the early changes observed after surgical menopause may not be attributable exclusively to the loss of ovarian function. Surgical stress, perioperative hemodynamic changes, inflammation, postoperative physical inactivity, and changes in volume status may also have influenced the 1-month echocardiographic findings. The absence of a non-menopausal surgical control group prevents separation of operation-related effects from those associated with surgical menopause. Nevertheless, the persistence of the 1-month time × group interaction after age adjustment suggests that the early difference warrants further investigation in studies specifically designed to distinguish hormonal from perioperative mechanisms.

From a clinical perspective, the present findings do not support treatment decisions based solely on an isolated reduction in LV GLS after surgical menopause. LV GLS may instead provide adjunctive information when interpreted together with symptoms, conventional echocardiographic findings, and established cardiovascular risk factors. Women undergoing surgical menopause may benefit from assessment and optimization of modifiable cardiovascular risk factors, consistent with recommendations emphasizing cardiovascular prevention during the menopausal transition [1]. Clinical decisions regarding hormone replacement therapy should not be based on LV GLS findings alone and should remain individualized according to gynecological indications, menopausal symptoms, age, time since menopause, and the overall benefit–risk profile.

The longer-term course of LV GLS after surgical menopause remains uncertain. The observed early deterioration may stabilize, recover, or progress over subsequent years. Previous studies have demonstrated that impaired LV GLS may be associated with adverse long-term cardiovascular outcomes even in populations with preserved LVEF [9]. However, the present study was not designed to evaluate clinical events or prognostic thresholds. Longer prospective follow-up is therefore required to determine whether the observed changes persist and whether they are associated with future cardiovascular morbidity.

Overall, our findings suggest that surgical menopause may be associated with a more pronounced early deterioration in LV GLS than natural menopause, particularly during the first month. However, the absence of a significant age-adjusted difference at 6 months, together with the lack of hormonal measurements and the potential influence of perioperative factors, prevents firm causal conclusions. These results should therefore be considered hypothesis-generating and require confirmation in larger, age-matched studies incorporating premenopausal and surgical control groups, serial hormonal measurements, and longer follow-up.

### Limitations

This study has several limitations. First, it was a single-center study with a relatively limited sample size and no formal a priori sample size calculation, which may restrict generalizability and reduce the power to detect smaller between-group differences, particularly at 6 months. Second, women in the surgical menopause group were older than those in the natural menopause group. Although age was incorporated into the adjusted mixed-effects model, residual age-related confounding cannot be excluded.

Third, circulating estradiol, follicle-stimulating hormone, and luteinizing hormone levels were not measured; therefore, the observed LV GLS changes cannot be directly attributed to hormonal alterations. The follow-up period was also limited to 6 months, precluding assessment of long-term cardiac outcomes and the persistence or prognostic significance of the observed changes.

Fourth, heart rate and blood pressure, including mean arterial pressure, were not systematically recorded at all echocardiographic visits. Individual baseline data on body mass index, lipid parameters, blood pressure, and smoking status were also not completely retained. Although patients with hypertension, diabetes mellitus, or hyperlipidemia requiring treatment were excluded and SCORE2 values were comparable between groups, the absence of these variables limits a comprehensive assessment of baseline comparability and loading conditions.

Fifth, speckle-tracking measurements may be affected by image quality, vendor-specific software, and technical variability. All analyses were performed by a single blinded experienced cardiologist, but formal intraobserver and interobserver reproducibility analyses were not conducted. Patients with inadequate image quality were excluded.

In addition, no age-matched premenopausal or non-menopausal surgical control group was included. The baseline menopausal stages were not biologically equivalent: women in the natural menopause group were assessed after retrospective confirmation of 12 months of amenorrhea, whereas those in the surgical group were assessed during the perimenopausal period before bilateral oophorectomy. This difference in timing and the potential influence of perioperative factors may have affected group comparability. Finally, a formal screening log was not maintained for women who declined participation or were excluded before enrollment, preventing reliable reconstruction of the total number screened and limiting assessment of potential selection bias.

## Conclusion

In this prospective echocardiographic follow-up study, surgical menopause was associated with a more pronounced early deterioration in LV GLS at 1 month compared with natural menopause, even after adjustment for age, despite preserved LVEF. However, the independent between-group difference was not maintained at 6 months after age adjustment. These findings suggest that speckle-tracking echocardiography may be useful as an adjunctive tool for detecting early subclinical changes in left ventricular longitudinal systolic function following surgical menopause. Nevertheless, the underlying mechanisms cannot be directly attributed to hormonal changes because circulating hormone levels were not measured. Larger multicenter studies including age-matched premenopausal controls, serial hormonal measurements, and longer follow-up are required to confirm these findings and determine their clinical and prognostic significance.

## Data Availability

The datasets generated and/or analyzed during the current study are not publicly available due to institutional restrictions and the confidentiality of patient data but are available from the corresponding author upon reasonable request.

## References

[1] El Khoudary SR, Aggarwal B, Beckie TM, Hodis HN, Johnson AE, Langer RD, Limacher MC, Manson JE, Stefanick ML, Allison MA, et al. Menopause Transition and Cardiovascular Disease Risk: Implications for Timing of Early Prevention: A Scientific Statement From the American Heart Association. Circulation. 2020;142:e506–32. 10.1161/CIR.0000000000000912. 33251828

[2] Yousefzai S, Amin Z, Faizan H, Ali M, Soni S, Friedman M, Kazmi A, Metlock FE, Sharma G, Javed Z. Cardiovascular Health During Menopause Transition: The Role of Traditional and Nontraditional Risk Factors. Methodist Debakey Cardiovasc J. 2025;21:121–8. 10.14797/mdcvj.1619. PMC12352403 40822367

[3] Ko SH, Kim HS. Menopause-associated lipid metabolic disorders and foods beneficial for postmenopausal women. Nutrients. 2020;12. 10.3390/nu12010202. PMC7019719 31941004

[4] Secosan C, Balint O, Pirtea L, Grigoras D, Balulescu L, Ilina R. Surgically induced menopause-a practical review of literature. Med (Kaunas). 2019;55. 10.3390/medicina55080482. PMC6722518 31416275

[5] Gumussoy S, Gumussoy M, Hortu I, Ergenoglu AM. The effect of surgical menopause after bilateral oophorectomy on hormonal changes, mucociliary clearance, and quality of life. Eur Arch Otorhinolaryngol. 2020;277:2793–800. 10.1007/s00405-020-06164-8. 32592009

[6] Cusimano MC, Chiu M, Ferguson SE, Moineddin R, Aktar S, Liu N, Baxter NN. Association of bilateral salpingo-oophorectomy with all cause and cause specific mortality: population based cohort study. BMJ. 2021;375:e067528. 10.1136/bmj-2021-067528. PMC8653240 34880044

[7] Attachaipanich T, Attachaipanich S, Kaewboot K. Cardiovascular outcomes of bilateral oophorectomy: A systematic review and meta-analysis. Indian Heart J. 2025;77:424–31. 10.1016/j.ihj.2025.08.001. PMC12793914 40812682

[8] Klaeboe LG, Edvardsen T. Echocardiographic assessment of left ventricular systolic function. J Echocardiogr. 2019;17:10–6. 10.1007/s12574-018-0405-5. 30390189

[9] Biering-Sorensen T, Biering-Sorensen SR, Olsen FJ, Sengelov M, Jorgensen PG, Mogelvang R, Shah AM, Jensen JS. Global longitudinal strain by echocardiography predicts long-term risk of cardiovascular morbidity and mortality in a low-risk general population: the copenhagen city heart study. Circ Cardiovasc Imaging. 2017;10. 10.1161/CIRCIMAGING.116.005521. PMC5363277 28264868

[10] Lang RM, Badano LP, Mor-Avi V, Afilalo J, Armstrong A, Ernande L, Flachskampf FA, Foster E, Goldstein SA, Kuznetsova T, et al. Recommendations for cardiac chamber quantification by echocardiography in adults: an update from the American Society of Echocardiography and the European Association of Cardiovascular Imaging. J Am Soc Echocardiogr. 2015;28:1–e3914. 10.1016/j.echo.2014.10.003. 25559473

[11] Nappi RE, Chedraui P, Lambrinoudaki I, Simoncini T. Menopause: a cardiometabolic transition. Lancet Diabetes Endocrinol. 2022;10:442–56. 10.1016/S2213-8587(22)00076-6. 35525259

[12] Xiang D, Liu Y, Zhou S, Zhou E, Wang Y. Protective Effects of Estrogen on Cardiovascular Disease Mediated by Oxidative Stress. Oxid Med Cell Longev. 2021;2021:5523516. 10.1155/2021/5523516. PMC8260319 34257804

[13] Wang YL, Yin LX, Li M. Assessment of left ventricular myocardial systolic dysfunction in premature ovarian insufficiency patients using echocardiographic layer-specific myocardial strain imaging. Echo Res Pract. 2024;11:20. 10.1186/s44156-024-00056-x. PMC11367989 39218983

